# Lyophilized rumen fluid from goats fed different roughage-based diets: fermentation characteristics, microbial communities, and post-reconstitution *in vitro* fermentation performance

**DOI:** 10.3389/fmicb.2026.1905439

**Published:** 2026-09-15

**Authors:** Meila Na, Wenliang Guo, Ran Zhang, Haidong Du, Kenan Li, Jing Zhang, Renhua Na

**Affiliations:** 1 College of Animal Science, Inner Mongolia Agricultural University, Hohhot, China; 2 College of Food and Agriculture, Hetao College, Bayannur, China; 3 College of Animal Science and Technology, Inner Mongolia Minzu University, Tongliao, China; 4 Institute of Grassland Research of Chinese Academy of Agricultural Sciences, Hohhot, China

**Keywords:** fermentation parameters, *in vitro* fermentation, lyophilized rumen fluid, roughage source, rumen fluid transplantation, rumen microbiota

## Abstract

**Introduction:**

Fresh rumen fluid is a promising donor material for rumen fluid transplantation (RFT), but poor storage stability and batch variability limit its use.

**Methods:**

In this study, lyophilized rumen fluid samples from goats fed alfalfa hay or corn stover diets were designated AHP and CSP, respectively. For the *in vitro* fermentation assay, portions of lyophilized rumen fluid from each batch within each dietary group were pooled and reconstituted; the substrate-supplemented groups were designated AH and CS, whereas AH-CON and CS-CON served as the corresponding substrate-free controls.

**Results:**

Lyophilization yields were 6.29 and 6.38%, and culturable carbohydrate-utilizing bacterial counts were 3.79 × 10⁷ and 3.93 × 10⁷ CFU/g, respectively, with no significant differences between AHP and CSP. AHP contained higher propionate and valerate concentrations (*p* < 0.05), whereas CSP contained higher acetate and isovalerate concentrations and had a higher acetate-to-propionate ratio (A/P; *p* < 0.05). Bacterial, fungal, and methanogen community structures differed significantly between groups (*p* < 0.01), and CSP showed higher bacterial and fungal Shannon indices (*p* < 0.01). AHP was significantly enriched in the bacterial genera *Xylanibacter*, *Segatella*, *Prevotellaceae_UCG-001*, and *Succinivibrio*; the fungal genera *Neocallimastix*, *Piromyces*, *Feramyces*, and *Aspergillus*; and the methanogen genera *Methanoculleus* and *Methanosphaera*. CSP was significantly enriched in the bacterial genera *norank_f__F082*, *Rikenellaceae_RC9_gut_group*, *norank_f__Bacteroidales_BS11_gut_group*, *norank_f__[Eubacterium]_coprostanoligenes_group*, *Ruminococcus*, *Monoglobus*, *norank_f__UCG-010*, and *Candidatus Saccharimonas*; the fungal genera *Orpinomyces*, *unclassified_f__Neocallimastigaceae*, *Cyllamyces*, *Caecomyces*, *Pecoramyces*, *Anaeromyces*, *Joblinomyces*, and *Alternaria*; and the methanogen genera *Methanobrevibacter* and *Methanolacinia*. Cross-domain correlation analysis showed predominantly positive associations among genera enriched within the same roughage-source group and negative associations between AHP- and CSP-enriched genera. Compared with their substrate-free controls, AH and CS showed increased gas production, bacterial crude protein (BCP), total volatile fatty acids (TVFA), acetate, propionate, and butyrate, and decreased pH (*p* < 0.05). CS showed higher pH, acetate, isovalerate, TVFA, and A/P, whereas AH showed higher gas production, butyrate, and valerate (*p* < 0.01).

**Discussion:**

Overall, the roughage source in the donor diets influenced the fermentation characteristics and microbial composition of lyophilized rumen fluid. After reconstitution, both inocula fermented exogenous substrate and exhibited different fermentation profiles, supporting their potential as storable inocula for standardization in RFT.

## 1 Introduction

Ruminal microbiota primarily consists of bacteria, archaea, fungi, and protozoa. Through synergistic interactions, these microorganisms secrete diverse digestive enzymes (e.g., cellulases and amylases) to degrade plant-based feedstuffs into host-utilizable metabolites such as volatile fatty acids (VFAs), ammonia nitrogen (NH₃–N), and bacterial crude protein (BCP) (Gao et al., 2024). Among these metabolites, VFAs supply approximately 60–70% of the energy required by ruminants and participate in epithelial development, energy metabolism, and immune regulation (Bergman, 1990; Akhtar et al., 2022). NH₃–N serves as a critical nitrogen source for rumen microbial protein synthesis, whereas BCP represents an important source of utilizable protein for ruminants (Huws et al., 2018). Therefore, modulating rumen microbial communities is a key strategy for improving rumen fermentation function in ruminants. This central role of the rumen microbiota has led to increasing interest in approaches that introduce or reshape microbial communities.

Rumen fluid transplantation (RFT) is a technique in which rumen fluid (including rumen microorganisms and their metabolites) from a specific donor is transferred into a recipient animal, aiming to reshape the recipient’s rumen microbial community, alter fermentation patterns, and optimize rumen function. Currently, RFT research has been applied to modulate gastrointestinal microbiota structure, improve rumen function, prevent and treat diseases, promote growth and development, and enhance milk production efficiency (Weimer et al., 2017; Mu et al., 2021; Li et al., 2023b; Chen et al., 2026). However, basic research on donor rumen fluid, as the core transplant material, remains relatively limited, which to some extent constrains in-depth investigation into the mechanisms of RFT.

Among the factors that determine the properties of donor rumen fluid, donor diet is particularly important. Roughage is an important component of the ruminant diet and plays an irreplaceable role in maintaining rumen function, microbial ecology, metabolic health, and production performance. Studies have shown that feeding high-fiber diets (e.g., Leymus chinensis, corn stover, or wheat straw) increases the relative abundance of *Fibrobacteres*, *Bacteroidetes*, and *Spirochaetes* within the rumen, whereas feeding high-protein or readily fermentable carbohydrate diets (e.g., alfalfa hay) increases the relative abundances of *Firmicutes* and *Proteobacteria* (Li et al., 2023a; Wang H. et al., 2023; Xie et al., 2025). Therefore, differences in rumen microenvironments shaped by roughage type may further influence the microbial composition and fermentation potential of donor rumen fluid, thereby affecting the functional properties of lyophilized inocula. Alfalfa hay and corn stover are two representative roughage sources in ruminant production in northern China. Alfalfa hay, a high-quality leguminous roughage, serves as an important source of premium fiber and protein in the diet (Zhu et al., 2013; Cao et al., 2016). Corn stover, a gramineous crop with abundant resources and favorable cost-effectiveness, represents the primary source of structural fiber in the basal diet of ruminants (Gao et al., 2021; Wang et al., 2021). In addition to donor diet, the form of transplantation may also play a role in determining the viability of the transplant.

At present, rumen fluid transplantation mainly relies on fresh rumen fluid. However, fresh rumen fluid is constrained by short shelf life, transportation difficulties, and poor batch-to-batch consistency, which limit its practical application in RFT. To improve the usability of donor rumen fluid, researchers have explored several preservation methods, including refrigeration, freezing, and lyophilization. Refrigeration at 4 °C is suitable for short-term transportation and storage for up to 72 h (Fabro et al., 2020), whereas freezing at −20 °C or −80 °C can extend the storage period (Tunkala et al., 2022; Zhao et al., 2024). The addition of cryoprotectants, such as glycerol or dimethyl sulfoxide, can alleviate freezing-induced damage (Denek et al., 2010; Zhao et al., 2024); however, the freeze–thaw process may still delay microbial recovery and reduce fermentation activity (Prates et al., 2010; Belanche et al., 2019). In contrast, lyophilization converts rumen fluid into a solid formulation that is more convenient for storage and transportation and has therefore attracted increasing attention as a rumen fluid preservation strategy (Nanthini et al., 2021; Simeonidis et al., 2025). Lyophilization can effectively preserve biological activity under low-temperature vacuum conditions and has been widely applied in microbial preservation and biopharmaceutical preparation (Abla and Mehanna, 2022; Islam et al., 2024; Uwineza and Zhang, 2026). In human fecal microbiota transplantation (FMT), lyophilized microbial formulations have demonstrated favorable stability and application potential (Zain et al., 2025). Therefore, applying lyophilization to rumen fluid preservation may offer a novel technical pathway for standardizing RFT donor materials. We hypothesized that lyophilization could preserve the basal microbial activity of rumen fluid, and that donor roughage source would yield lyophilized inocula with distinct microbial communities and fermentation phenotypes. Based on this, the present study aimed to prepare lyophilized rumen fluid from goats fed alfalfa hay- or corn stover-based diets and to evaluate their lyophilization yields, culturable carbohydrate-utilizing bacterial counts, fermentation parameters, microbial community composition, predicted metabolic potential, and post-reconstitution *in vitro* fermentation performance, thereby providing a basis for RFT donor material development and subsequent *in vivo* transplantation research.

## 2 Materials and methods

### 2.1 Donor animals, feeding, and preparation of lyophilized rumen fluid

All procedures involving the donor goats and rumen fluid collection were approved by the Animal Welfare and Ethics Committee of Inner Mongolia Agricultural University (Approval No: NND2024160).

Forty healthy, non-pregnant Inner Mongolian Cashmere goats (38 months old; 37.28 ± 3.32 kg BW) were selected from Jinlai Cashmere Goat Farm (Hohhot, China), which operates under an established animal-health and biosecurity program for major infectious diseases, including brucellosis and foot-and-mouth disease. All candidate donor goats were individually confirmed negative for brucellosis by serological testing during donor selection and again before the first rumen fluid collection. The goats were randomly assigned to two dietary groups (*n* = 20 per group) and fed isoenergetic and isonitrogenous diets containing either alfalfa hay or corn stover as the sole roughage source. The forage-to-concentrate ratio was 50:50. Diets were formulated according to the Nutrient Requirements of Cashmere Goats (NY/T 4048-2021) (Ministry of Agriculture and Rural Affairs of the People’s Republic of China, 2021), and the diet composition and nutrient levels are shown in Supplementary Table S1. Goats in each group were housed in two separate earthen-floored pens equipped with automatic drinking systems, with 10 goats per pen. The feeding trial lasted 60 days, during which goats were fed twice daily at 08:00 and 16:00 and had ad libitum access to feed and water. The daily feed allowance was adjusted to maintain refusals of at least 5%.

Rumen fluid was collected from the donor goats via oral intubation at 3 h after the morning feeding, beginning on day 30 of the feeding trial. Samples were collected at 5-d intervals on six occasions. For each goat, the first 100 mL of rumen fluid was discarded to minimize saliva contamination, and the subsequent 100 mL was retained. Immediately after collection, rumen fluid from each goat was filtered separately through four layers of cheesecloth under a continuous CO₂ stream. The filtered rumen fluid from all goats within the same dietary group was then pooled and thoroughly homogenized to form one group-specific rumen fluid batch. Thus, one pooled rumen fluid batch was generated for each dietary group at each collection. Each pooled rumen fluid batch was mixed with a 10% (w/v) skim milk solution at a 1:1 (v/v) ratio, flushed with CO₂, sealed, and pre-frozen at −80 °C. The frozen mixtures were subsequently lyophilized using a vacuum freeze-dryer (GZL-5, Beijing Songyuan Huaxing Technology Development Co., Ltd., Beijing, China). The lyophilized rumen fluid powders prepared from rumen fluid collected from goats fed an alfalfa hay diet or a corn stover diet were designated AHP and CSP, respectively. Because rumen fluid was collected and lyophilized independently on six occasions, six independent batches of lyophilized rumen fluid were obtained for each dietary group. Lyophilization yield was calculated separately for each batch. After lyophilization, a portion of each batch of lyophilized rumen fluid was divided into aliquots according to the intended analysis. Aliquots intended for microbial sequencing were stored at −80 °C until DNA extraction, whereas those intended for culturable carbohydrate-utilizing bacterial enumeration and fermentation parameter measurements were stored at −20 °C until analysis. For these analyses, each independently prepared batch of lyophilized rumen fluid was considered the experimental unit, resulting in six batch-level replicates per dietary group (*n* = 6). After subsampling for the above analyses, the remaining material from the six batches within each dietary group was combined and thoroughly homogenized to form one pooled preparation per dietary group. Each pooled preparation was aliquoted and stored at −20 °C until use in the subsequent *in vitro* fermentation assay.

### 2.2 Lyophilization yield, culturable carbohydrate-utilizing bacterial counts, and fermentation parameters of lyophilized rumen fluid

Lyophilization yield was calculated separately for each batch. Culturable carbohydrate-utilizing bacterial counts were determined using a carbohydrate-based anaerobic medium (Leedle and Hespell, 1980).

Before reconstitution, the stored aliquots of lyophilized rumen fluid were allowed to equilibrate to room temperature while sealed. The aliquots were then reconstituted in sterile phosphate-buffered saline (PBS) at a 1:10 (w/v) ratio, and the pH of the suspension was measured using a portable pH meter (Starter 300; Ohaus Instruments Co., Ltd., Shanghai, China). The suspension was then centrifuged at 3000 × g for 10 min at 4 °C (KL04A, Kaida, Changsha, China), and the supernatant was collected for the determination of fermentation parameters and enzyme activities. NH₃–N concentration was determined using the phenol-hypochlorite colorimetric method (Winichayakul et al., 2020). Volatile fatty acids (VFAs) were quantified by gas chromatography (GC-2030, Shimadzu, Japan) using 2-ethylbutyric acid as the internal standard (Lin et al., 2019). Bacterial crude protein (BCP) content was measured using the Coomassie Brilliant Blue method (Chanjula and Cherdthong, 2018). Cellulase activity was determined using an enzymatic colorimetric kit (Solarbio, Beijing, China), and α-amylase activity was measured using an iodine-starch colorimetric kit (Nanjing Jiancheng Bioengineering Institute, Nanjing, China).

### 2.3 Microbial DNA extraction, sequencing, and bioinformatic analysis

Microbial DNA was extracted from the lyophilized rumen fluid samples using the E.Z.N.A.® Soil DNA Kit (Omega Bio-tek, Norcross, GA, USA) according to the manufacturer’s instructions. DNA integrity was assessed by agarose gel electrophoresis, and DNA concentration and purity were determined using a NanoDrop 2000 spectrophotometer (Thermo Fisher Scientific, Waltham, MA, USA). The bacterial 16S rRNA gene V3–V4 region, fungal ITS1 region, and methanogen mcrA gene were amplified and sequenced on an Illumina NextSeq 2000 platform.

Raw paired-end sequencing reads were quality-filtered using fastp (version 0.23.4) and merged using FLASH (version 1.2.11). Denoising was performed using the DADA2 plugin within the QIIME 2 pipeline. Taxonomic classification was conducted against the SILVA database (version 138.2) for bacteria, UNITE database (version 9.0) for fungi, and FunGene database (version 9.6) for methanogens, with a confidence threshold of 70%. Alpha-diversity indices were calculated, and beta diversity was assessed based on Bray–Curtis dissimilarities. Principal coordinate analysis (PCoA) combined with ANOSIM was employed to evaluate community dissimilarities between groups. Differentially abundant genera were identified using LEfSe analysis (LDA score > 4.0, *p* < 0.05). Spearman correlation analysis was performed to explore correlations among genera and their associations with fermentation parameters, with |r| > 0.6 and *p* < 0.05 considered significant after Benjamini–Hochberg correction. Bacterial functional prediction was performed using PICRUSt2 (version 2.2.0) based on 16S rRNA gene sequences. All bioinformatic analyses were conducted on the Majorbio Cloud Platform^1^. Detailed primer sequences, PCR amplification conditions, and library preparation procedures are provided in Supplementary methods S1.

### 2.4 *In vitro* fermentation performance of reconstituted lyophilized rumen fluid

A completely randomized design was used for the *in vitro* fermentation assay. The pooled lyophilized rumen fluid preparations from the two dietary groups, as described in Section 2.1, were used to prepare the fermentation inocula. Four treatments were established: AH, consisting of the reconstituted pooled preparation of lyophilized rumen fluid based on an alfalfa hay diet, with 1 g of substrate; AH-CON, consisting of the corresponding reconstituted preparation without substrate; CS, consisting of the reconstituted pooled preparation of lyophilized rumen fluid based on a corn stover diet, with 1 g of substrate; and CS-CON, consisting of the corresponding reconstituted preparation without substrate. AH-CON and CS-CON served as substrate-free controls. Each treatment was performed in triplicate within each run, and the experiment was independently repeated across three runs, giving nine replicates per treatment (*n* = 9).

The fermentation substrate was ground to pass through a 1-mm screen, and its composition and nutrient levels are shown in Supplementary Table S2. Before reconstitution, the stored aliquots of lyophilized rumen fluid were allowed to equilibrate to room temperature while sealed. The aliquots were then reconstituted in sterile distilled water at a 1:10 (w/v) ratio, and the resulting suspensions were pre-incubated at 39 °C under continuous CO₂ flushing. The reconstituted fluids were then mixed with anaerobic artificial saliva prepared according to Menke and Steingass (1988) at a 1:2 (v/v) ratio to prepare artificial fermentation fluids. For each incubation, 60 mL of fermentation fluid was dispensed into 100-mL fermentation bottles according to the treatment design. The bottles were flushed with CO₂, sealed, and incubated at 39 °C for 24 h using an automated gas recording system (ANKOM AGRS, ANKOM Technology, Macedon, NY, USA). Gas production was recorded automatically throughout incubation. At the end of fermentation, bottles were immediately placed in an ice bath to terminate microbial activity. The pH was measured, and the fermentation fluid was centrifuged to collect the supernatant for the determination of NH₃–N, VFAs, and BCP as described in Section 2.2.

### 2.5 Statistical analysis

Data were analyzed using SAS 9.4 (SAS Institute Inc., Cary, NC, USA). Lyophilization yield, culturable carbohydrate-utilizing bacterial counts, and fermentation parameters were compared between the two dietary groups using Student’s *t*-test. Post-reconstitution *in vitro* fermentation data were analyzed using one-way ANOVA followed by Duncan’s multiple range test. Data are presented as means ± standard deviation (SD), and statistical significance was declared at *p* < 0.05.

## 3 Results

### 3.1 Lyophilization yield, culturable carbohydrate-utilizing bacterial counts, and fermentation characteristics of lyophilized rumen fluid

As shown in Table 1, lyophilization yield and culturable carbohydrate-utilizing bacterial counts did not differ significantly between the AHP and CSP groups (*p* > 0.05). The AHP group had significantly higher concentrations of propionate (*p* = 0.0226) and valerate (*p* = 0.0002), whereas the CSP group had significantly higher concentrations of acetate (*p* = 0.0422) and isovalerate (*p* = 0.0298), as well as a higher acetate-to-propionate ratio (A/P; *p* < 0.0001).

**Table 1:** Lyophilization yield, culturable carbohydrate-utilizing bacterial counts, and fermentation parameters of lyophilized rumen fluid based on different roughage sources.

Items	AHP	CSP	*p*-value
Lyophilization yield (%)	6.29 ± 0.18	6.38 ± 0.14	0.4211
Culturable bacterial count (×10^7^ CFU/g)	3.79 ± 0.89	3.93 ± 0.83	0.7737
pH	6.23 ± 0.01	6.24 ± 0.01	0.0571
NH_3_-N (mg/g)	1.67 ± 0.07	1.74 ± 0.06	0.1108
BCP (mg/g)	3.50 ± 0.14	3.38 ± 0.15	0.1911
Total VFA (mmol/100 g)	76.62 ± 8.05	79.22 ± 6.04	0.5762
Acetic (mmol/100 g)	52.06 ± 5.09	58.75 ± 3.92	0.0422
Propionic (mmol/100 g)	15.00 ± 1.91	12.13 ± 1.44	0.0226
Butyric (mmol/100 g)	8.15 ± 1.09	7.17 ± 0.99	0.1659
Isobutyric (mmol/100 g)	0.26 ± 0.03	0.25 ± 0.03	0.7434
Valeric (mmol/100 g)	0.77 ± 0.11	0.44 ± 0.06	0.0002
Isovaleric (mmol/100 g)	0.37 ± 0.05	0.47 ± 0.07	0.0298
A/P	3.49 ± 0.17	4.88 ± 0.39	<0.0001
Cellulase (U/mg)	5.02 ± 0.34	5.15 ± 0.38	0.5805
α-Amylase (U/g)	31.57 ± 1.96	30.43 ± 2.17	0.4035

Values are presented as means ± SD (*n* = 6). VFA contents are expressed as mmol/100 g of lyophilized powder. AHP, lyophilized rumen fluid based on the alfalfa hay diet; CSP, lyophilized rumen fluid based on the corn stover diet. NH₃–N, ammonia nitrogen; BCP, bacterial crude protein; VFAs, volatile fatty acids; A/P, acetate-to-propionate ratio. Differences were considered significant at *p* < 0.05.

### 3.2 Microbial community profiles of lyophilized rumen fluid based on different donor roughage sources

#### 3.2.1 Microbial α- and β-diversity

PCoA demonstrated distinct clustering of bacterial, fungal, and methanogen communities between the AHP and CSP groups (ANOSIM: *R* = 1.0000, *p* = 0.0030; Figures 1A–C). Chao indices did not differ significantly between groups for any microbial community (*p* > 0.05; Figures 1D–F), whereas Shannon indices were significantly higher in the CSP group for bacteria and fungi (*p* < 0.01; Figures 1G,H), with no significant difference observed for methanogens (*p* > 0.05; Figure 1I).

**Figure 1:**
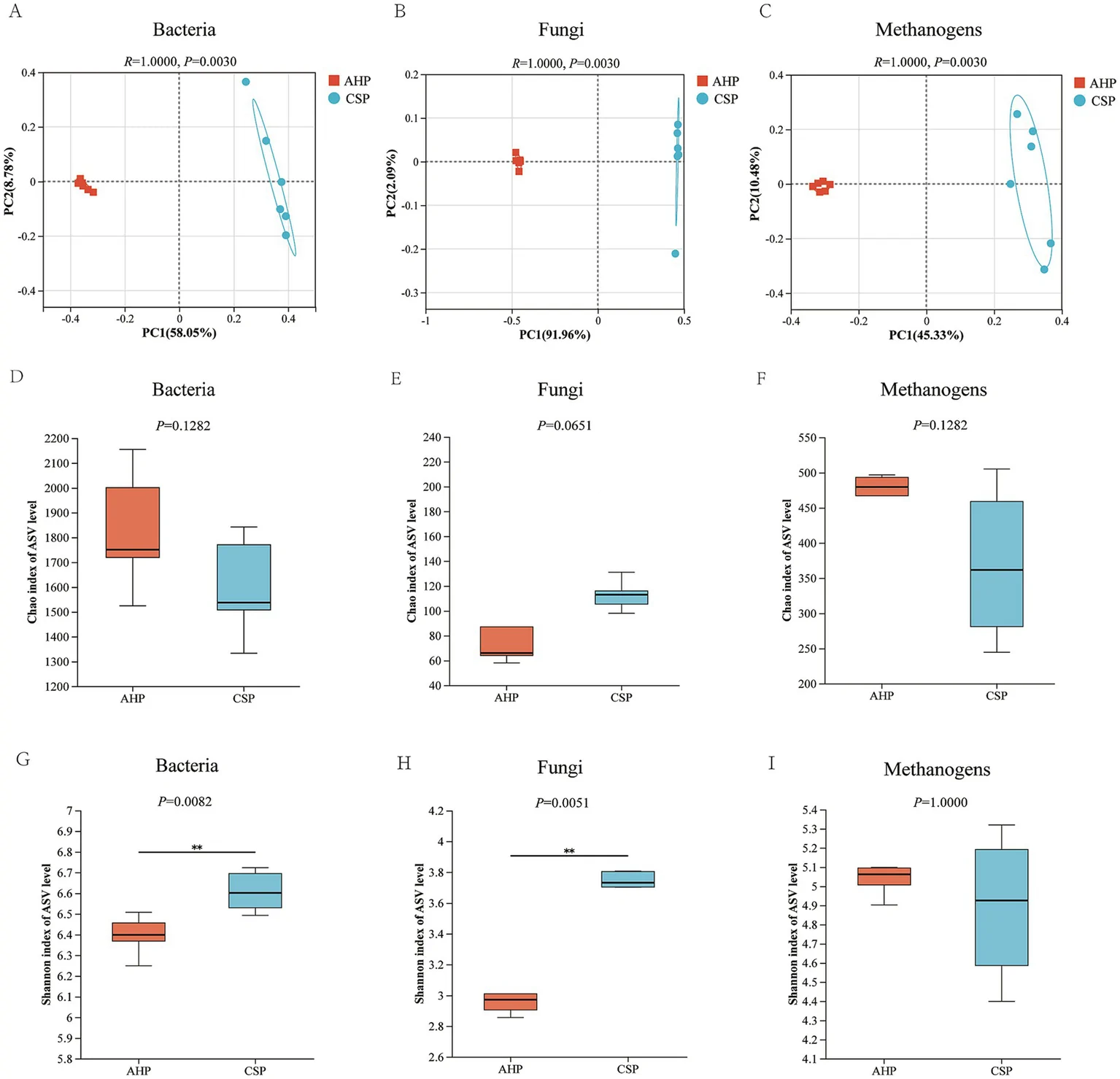
PCoA plots and α-diversity indices of bacterial, fungal, and methanogen communities in lyophilized rumen fluid based on different roughage sources. PCoA plots were constructed based on Bray–Curtis dissimilarities for bacterial **(A)**, fungal **(B)**, and methanogen **(C)** communities. Chao indices of bacterial **(D)**, fungal **(E)**, and methanogen **(F)** communities. Shannon indices of bacterial **(G)**, fungal **(H)**, and methanogen **(I)** communities. AHP, lyophilized rumen fluid based on the alfalfa hay diet; CSP, lyophilized rumen fluid based on the corn stover diet. Asterisks indicate significant differences between groups (**p < 0.01).

#### 3.2.2 Bacterial composition

16S rRNA sequencing detected a total of 21 bacterial phyla and 295 genera across all samples. *Bacteroidota* and *Bacillota* were the dominant phyla shared by both groups (Figure 2A). At the genus level, *Xylanibacter* was the most abundant genus in both groups, followed by *norank_f__Bacteroidales_RF16_group* in the AHP group and *norank_f__F082* in the CSP group (Figure 2B). LEfSe analysis revealed that *Succinivibrio*, *Xylanibacter*, *Segatella*, and *Prevotellaceae_UCG-001* were enriched in the AHP group, whereas *norank_f__F082*, *Rikenellaceae_RC9_gut_group*, *norank_f__Bacteroidales_BS11_gut_group*, *norank_f__[Eubacterium]_coprostanoligenes_group*, *Ruminococcus*, *Monoglobus*, *norank_f__UCG-010*, and *Candidatus Saccharimonas* were enriched in the CSP group (LDA score > 4, *p* < 0.05; Figure 2C). Spearman correlation analysis further revealed associations between these differentially enriched genera and fermentation indices (Figure 2D). pH was significantly positively correlated with *Rikenellaceae_RC9_gut_group*, *Ruminococcus*, *norank_f__UCG-010*, and *Candidatus Saccharimonas*, and significantly negatively correlated with *Succinivibrio*. Acetate was significantly positively correlated with *Rikenellaceae_RC9_gut_group*, *norank_f__[Eubacterium]_coprostanoligenes_group*, *Monoglobus*, and *Candidatus Saccharimonas*, and significantly negatively correlated with *Succinivibrio* and *Segatella*. Propionate was significantly positively correlated with *Prevotellaceae_UCG-001* and *Segatella*, and significantly negatively correlated with *Rikenellaceae_RC9_gut_group*, *Ruminococcus*, *norank_f__F082*, *norank_f__Bacteroidales_BS11_gut_group*, *Monoglobus*, *norank_f__UCG-010*, and *Candidatus Saccharimonas*. Butyrate was significantly positively correlated with *Prevotellaceae_UCG-001* and significantly negatively correlated with *norank_f__F082*. Isovalerate was significantly positively correlated with *norank_f__[Eubacterium]_coprostanoligenes_group* and significantly negatively correlated with *Succinivibrio*. Valerate was significantly positively correlated with *Prevotellaceae_UCG-001*, *Succinivibrio*, *Segatella*, and *Xylanibacter*, and significantly negatively correlated with *Rikenellaceae_RC9_gut_group*, *Ruminococcus*, *norank_f__F082*, *norank_f__[Eubacterium]_coprostanoligenes_group*, *norank_f__Bacteroidales_BS11_gut_group*, *Monoglobus*, *norank_f__UCG-010*, and *Candidatus Saccharimonas*. A/P exhibited an inverse trend relative to valerate.

**Figure 2:**
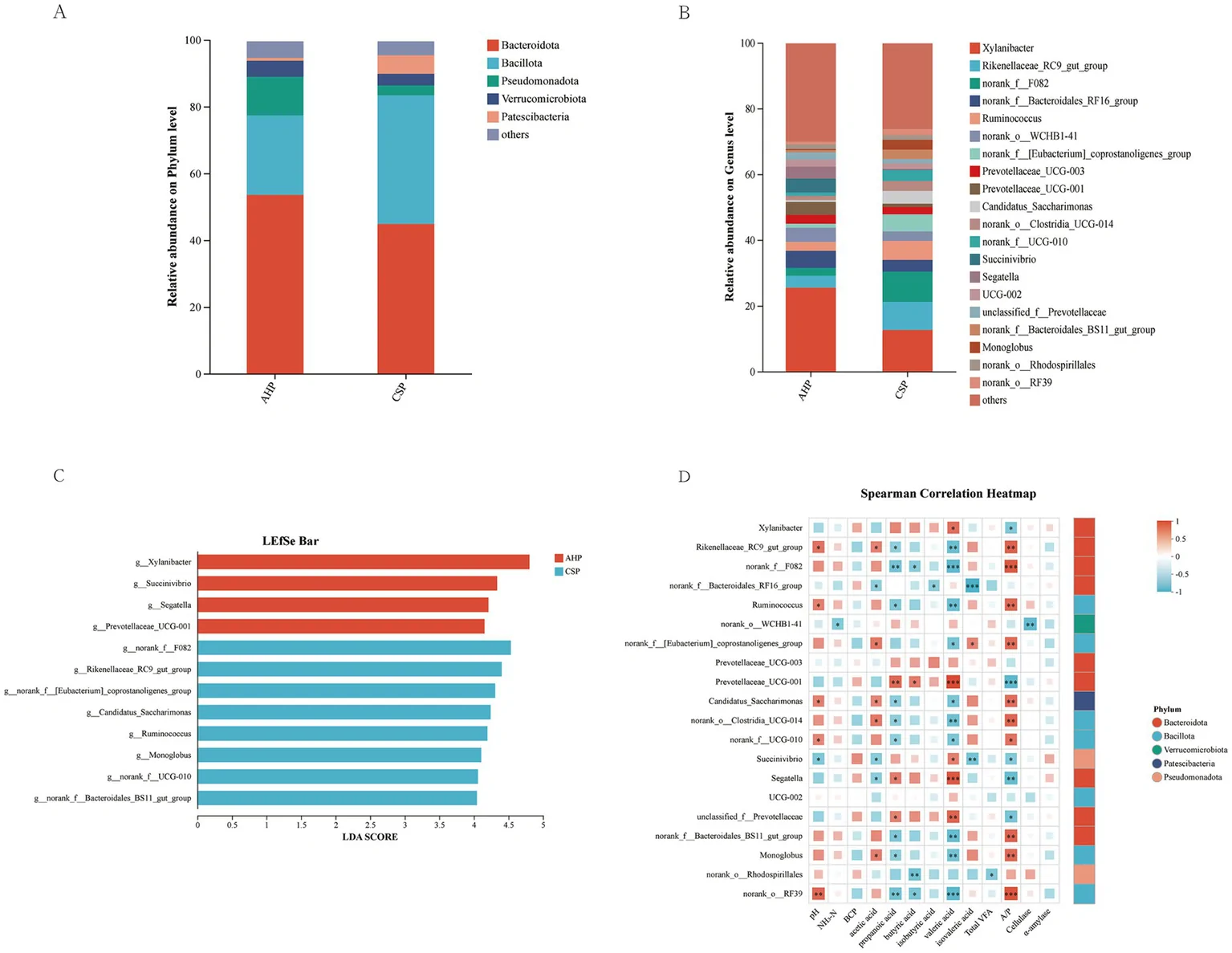
Bacterial community composition and correlations between bacterial genera and fermentation parameters in lyophilized rumen fluid based on different roughage sources. Relative abundances at the phylum **(A)** and genus **(B)** levels. Differentially abundant bacterial genera identified by LEfSe analysis (**C**; LDA score > 4.0, *p* < 0.05). Spearman correlation heatmap between the top 20 bacterial genera and fermentation parameters **(D)**. In panel **D**, red and blue indicate positive and negative correlations, respectively. AHP, lyophilized rumen fluid based on the alfalfa hay diet; CSP, lyophilized rumen fluid based on the corn stover diet. Asterisks indicate significant correlations (*p < 0.05; **p < 0.01; ***p < 0.001).

#### 3.2.3 Fungal composition

ITS sequencing identified a total of eight fungal phyla and 136 genera across the lyophilized rumen fluid samples. *Neocallimastigomycota* was dominant in both the AHP and CSP groups, followed by *Ascomycota* (Figure 3A). At the genus level, the dominant genera in the AHP group were *Neocallimastix* and *Piromyces*, whereas those in the CSP group were *Orpinomyces* and *Piromyces* (Figure 3B). LEfSe analysis revealed that *Neocallimastix*, *Piromyces*, *Feramyces*, *Aspergillus*, and *Fungi_gen_Incertae_sedis* were enriched in the AHP group, while *Orpinomyces*, *unclassified_f__Neocallimastigaceae*, *Cyllamyces*, *Caecomyces*, *Pecoramyces*, *Anaeromyces*, *Joblinomyces*, and *Alternaria* were enriched in the CSP group (LDA score > 4, *p* < 0.05; Figure 3C). Correlation analysis further revealed associations between these differentially enriched fungal genera and fermentation indices (Figure 3D). pH was significantly positively correlated with *Pecoramyces* and *unclassified_f__Neocallimastigaceae*, and significantly negatively correlated with *Neocallimastix*. Acetate was significantly positively correlated with *Cyllamyces*, *Caecomyces*, *Anaeromyces*, and *Joblinomyces*, and significantly negatively correlated with *Feramyces*. Propionate was significantly positively correlated with *Piromyces* and *Feramyces*, and significantly negatively correlated with *Orpinomyces*, *Pecoramyces*, and *unclassified_f__Neocallimastigaceae*. Butyrate was significantly negatively correlated with *Orpinomyces* and *Pecoramyces*. Isovalerate was significantly positively correlated with *Caecomyces*, *Anaeromyces*, and *Joblinomyces*, and significantly negatively correlated with *Neocallimastix*, *Feramyces*, and *Aspergillus*. Valerate was significantly positively correlated with *Piromyces*, *Neocallimastix*, *Feramyces*, and *Aspergillus*, and significantly negatively correlated with *Orpinomyces*, *Cyllamyces*, *Caecomyces*, *Anaeromyces*, *Joblinomyces*, *Pecoramyces*, and *unclassified_f__Neocallimastigaceae*. A/P exhibited an inverse trend relative to valerate.

**Figure 3:**
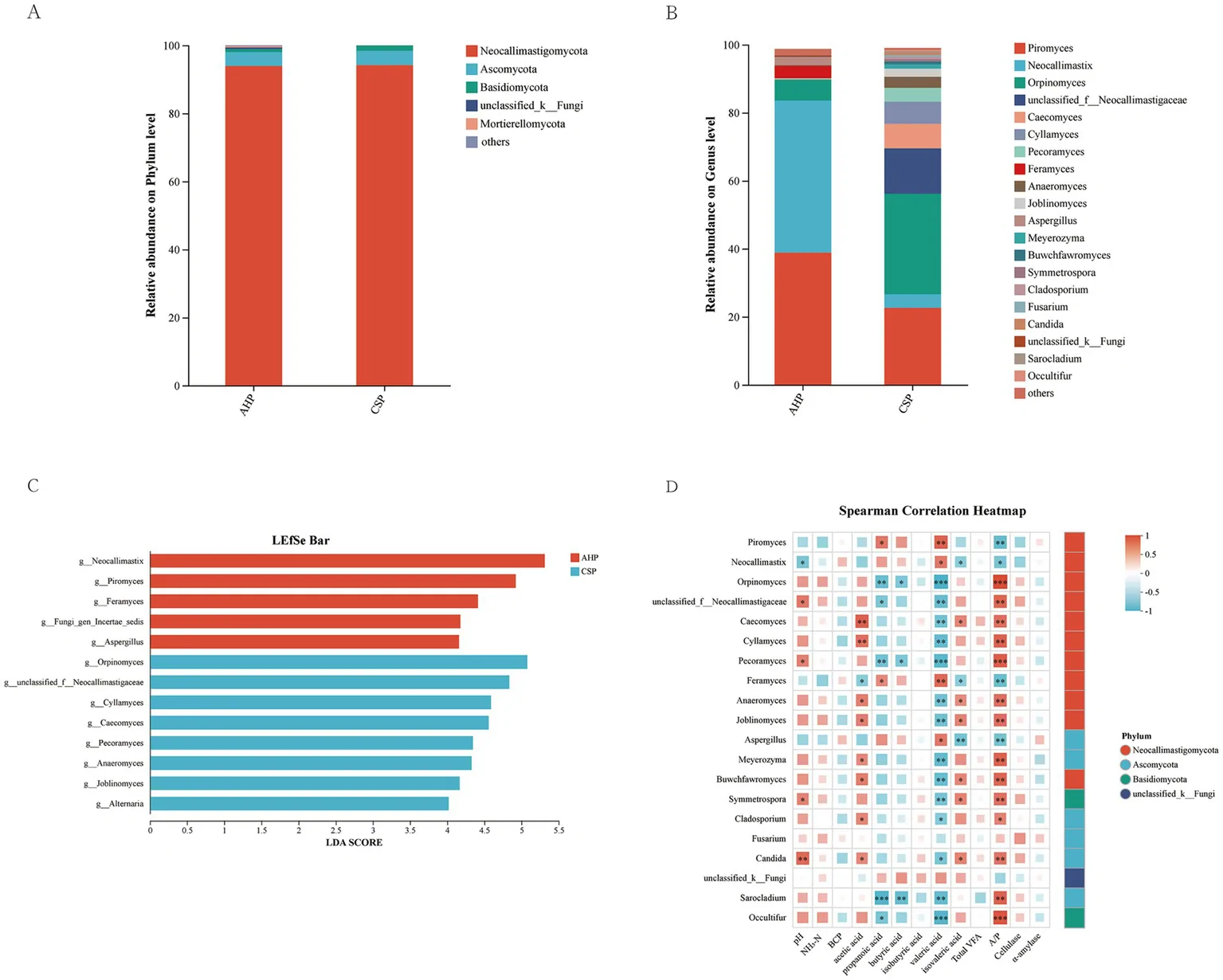
Fungal community composition and correlations between fungal genera and fermentation parameters in lyophilized rumen fluid based on different roughage sources. Relative abundances at the phylum **(A)** and genus **(B)** levels. Differentially abundant fungal genera identified by LEfSe analysis (**C**; LDA score > 4.0, *p* < 0.05). Spearman correlation heatmap between the top 20 fungal genera and fermentation parameters **(D)**. In panel **D**, red and blue indicate positive and negative correlations, respectively. AHP, lyophilized rumen fluid based on the alfalfa hay diet; CSP, lyophilized rumen fluid based on the corn stover diet. Asterisks indicate significant correlations (*p < 0.05; **p < 0.01; ***p < 0.001).

#### 3.2.4 Methanogenic composition

mcrA gene sequencing identified a total of four methanogen phyla and 18 genera across the lyophilized rumen fluid samples from the AHP and CSP groups. *Euryarchaeota* was the dominant phylum in both groups (Figure 4A). At the genus level, *Methanobrevibacter* and *Methanoculleus* were the dominant genera in both groups (Figure 4B). LEfSe analysis revealed that *Methanoculleus* and *Methanosphaera* were enriched in the AHP group, whereas *Methanobrevibacter* and *Methanolacinia* were enriched in the CSP group (LDA score > 4, *p* < 0.05; Figure 4C). Spearman correlation analysis further revealed associations between these differentially enriched methanogen genera and fermentation indices (Figure 4D). pH was significantly positively correlated with *Methanolacinia*. Acetate was significantly positively correlated with *Methanolacinia* and significantly negatively correlated with *Methanosphaera*. Propionate was significantly positively correlated with *Methanoculleus*. Isovalerate was significantly positively correlated with *Methanolacinia*. Cellulase activity was significantly positively correlated with *Methanobrevibacter*. Valerate was significantly positively correlated with *Methanoculleus* and *Methanosphaera*, and significantly negatively correlated with *Methanobrevibacter* and *Methanolacinia*. A/P exhibited an inverse trend relative to valerate.

**Figure 4:**
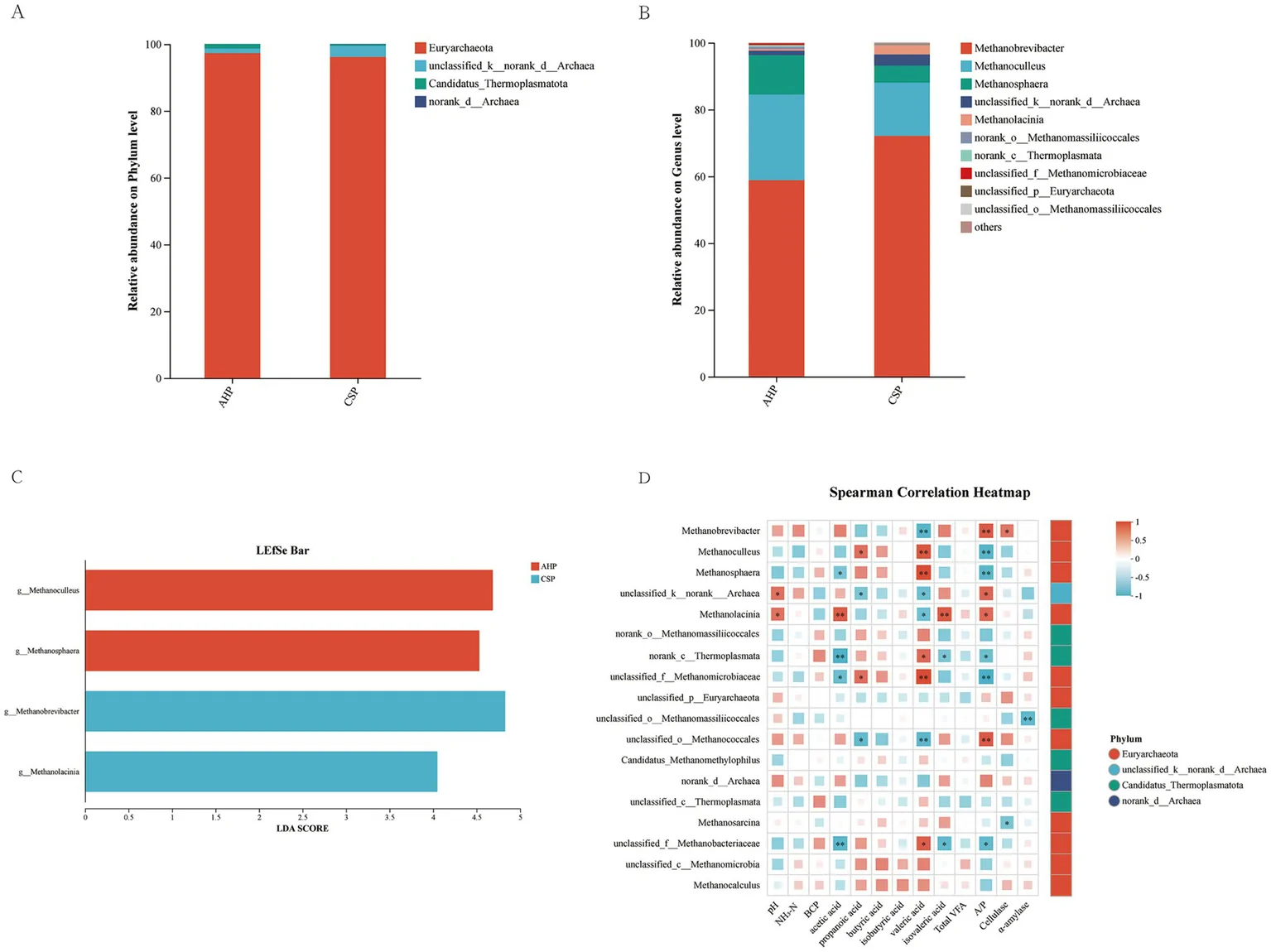
Methanogen community composition and correlations between methanogen genera and fermentation parameters in lyophilized rumen fluid based on different roughage sources. Relative abundances at the phylum **(A)** and genus **(B)** levels. Differentially abundant methanogen genera identified by LEfSe analysis (**C**; LDA score > 4.0, *p* < 0.05). Spearman correlation heatmap between methanogen genera and fermentation parameters **(D)**. In panel **D**, red and blue indicate positive and negative correlations, respectively. AHP, lyophilized rumen fluid based on the alfalfa hay diet; CSP, lyophilized rumen fluid based on the corn stover diet. Asterisks indicate significant correlations (*p < 0.05; **p < 0.01).

#### 3.2.5 Correlations within and among bacterial, fungal, and methanogen communities

Spearman correlation analysis of the top 20 bacterial, fungal, and methanogen genera revealed distinct cross-domain association patterns (Figure 5). AHP-enriched bacterial genera, including *Prevotellaceae_UCG-001*, *Succinivibrio*, *Segatella*, and *Xylanibacter*, were generally positively correlated with AHP-enriched fungi, including *Neocallimastix*, *Piromyces*, *Feramyces*, and *Aspergillus*, as well as AHP-enriched methanogens such as *Methanoculleus* and *Methanosphaera*. Among these, *Xylanibacter* showed no significant correlation with *Neocallimastix* or *Piromyces*, whereas all remaining pairwise correlations were significantly positive. In contrast, CSP-enriched bacterial genera, including *Rikenellaceae_RC9_gut_group*, *norank_f__F082*, *Ruminococcus*, *norank_f__[Eubacterium]_coprostanoligenes_group*, *Candidatus Saccharimonas*, *norank_f__UCG-010*, *norank_f__Bacteroidales_BS11_gut_group*, and *Monoglobus*, were generally positively correlated with CSP-enriched fungi, including *Orpinomyces*, *Cyllamyces*, *Caecomyces*, *Pecoramyces*, *Anaeromyces*, and *Joblinomyces*, as well as CSP-enriched methanogens such as *Methanobrevibacter* and *Methanolacinia*. Among these, *Methanobrevibacter* showed no significant correlation with *norank_f__[Eubacterium]_coprostanoligenes_group*, *norank_f__UCG-010*, or *Monoglobus*, whereas all remaining pairwise correlations were significantly positive. Between the AHP and CSP groups, enriched genera were negatively correlated; however, no significant negative correlation was observed between *Xylanibacter* and *Cyllamyces*, or between *norank_f__UCG-010* and *Methanoculleus*, whereas all other pairwise intergroup correlations were significantly negative.

**Figure 5:**
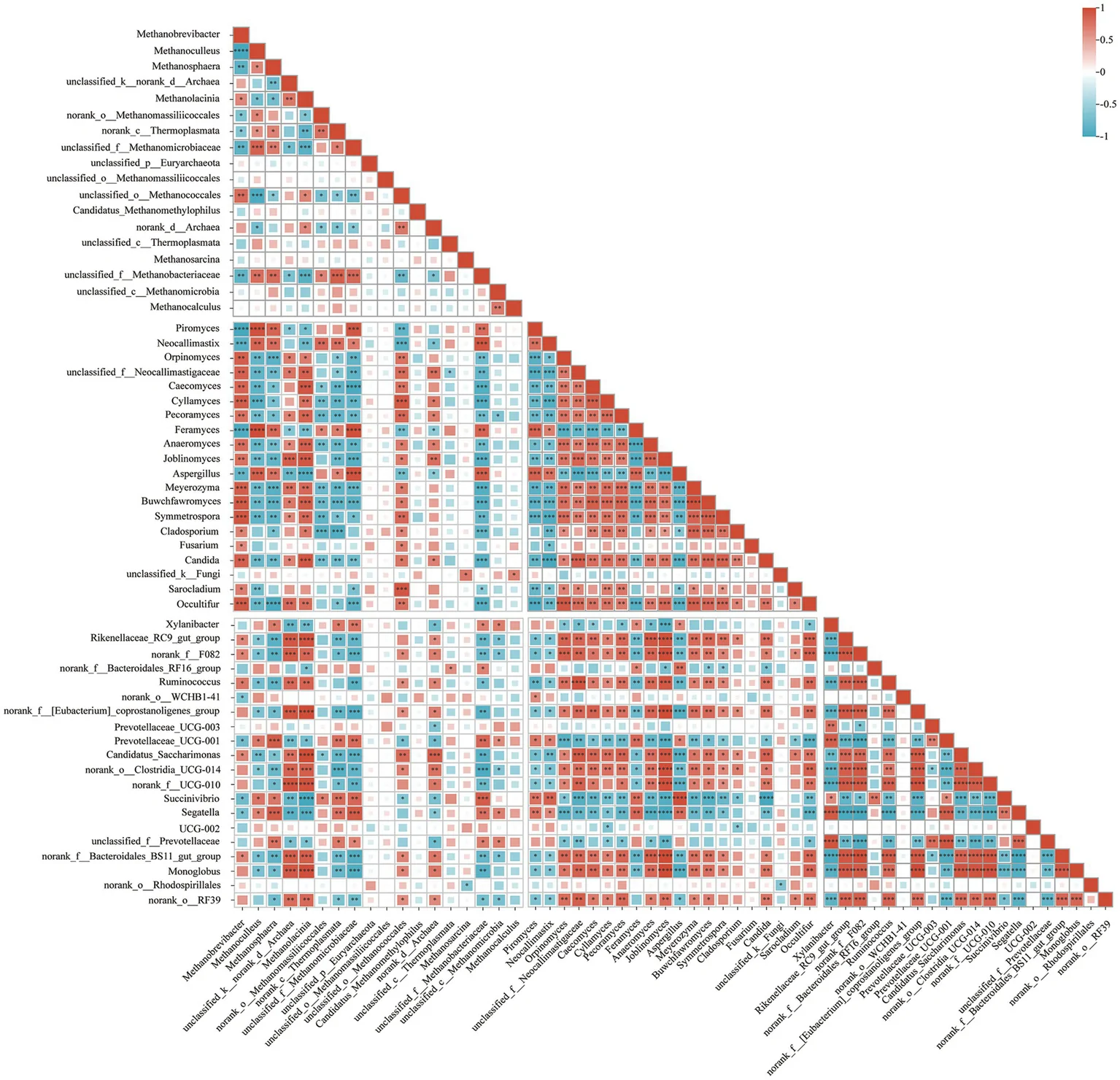
Correlations within and among bacterial, fungal, and methanogen communities in lyophilized rumen fluid based on different roughage sources. Spearman correlation heatmap showing pairwise associations among the top 20 bacterial, fungal, and methanogen genera. The lower triangular matrix displays correlation coefficients (*r*), with red and blue indicating positive and negative correlations, respectively. Asterisks indicate significant correlations (*p < 0.05; **p < 0.01; ***p < 0.001; ****p < 0.0001).

#### 3.2.6 PICRUSt2-based prediction of bacterial functions

PICRUSt2-based KEGG functional prediction showed that Metabolism was the predominant Level 1 category in both groups, accounting for >77% of predicted functional genes, with no significant difference between the AHP and CSP groups (Figure 6A). At Level 2, Global and overview maps, Carbohydrate metabolism, and Amino acid metabolism were the most abundant functional categories in both groups, each exceeding 5% relative abundance. Carbohydrate metabolism was significantly enriched in the AHP group, whereas Amino acid metabolism was significantly higher in the CSP group (Figure 6B).

**Figure 6:**
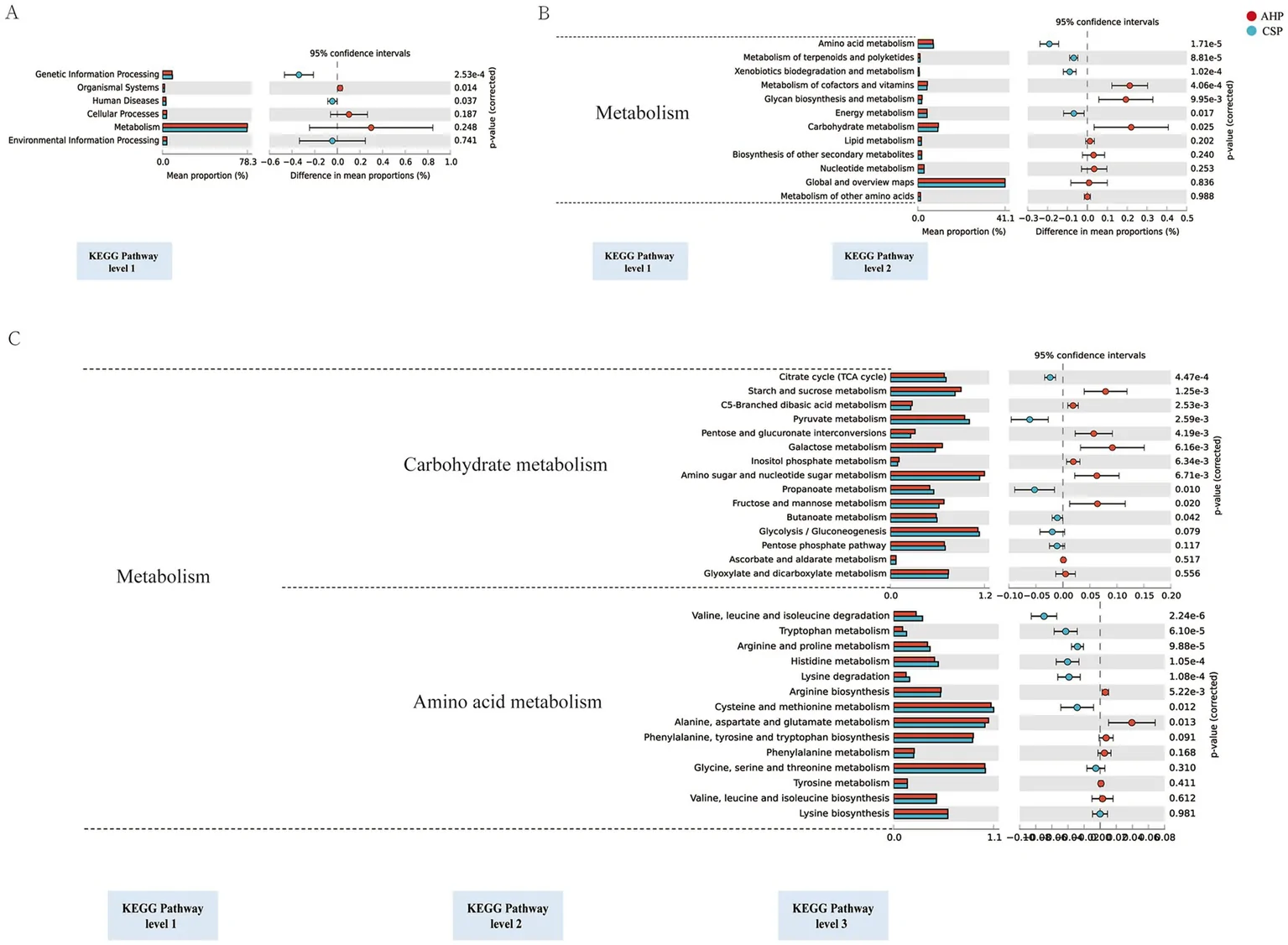
PICRUSt2-based prediction of bacterial functional profiles in lyophilized rumen fluid based on different roughage sources. Predicted KEGG pathways at Level 1 **(A)**, Level 2 within the Metabolism category **(B)**, and Level 3 pathways within Carbohydrate metabolism and Amino acid metabolism, the two Level 2 categories that differed significantly between groups and each had a mean relative abundance > 5% **(C)**. Error bars indicate 95% confidence intervals. Statistical significance was assessed using Welch’s *t*-test with Benjamini–Hochberg FDR correction. AHP, lyophilized rumen fluid based on the alfalfa hay diet; CSP, lyophilized rumen fluid based on the corn stover diet.

At Level 3, multiple pathways within Carbohydrate metabolism and Amino acid metabolism differed significantly between the two groups (Figure 6C). Within Carbohydrate metabolism, the AHP group showed higher relative abundances of Starch and sucrose metabolism, C5-Branched dibasic acid metabolism, Pentose and glucuronate interconversions, Galactose metabolism, Inositol phosphate metabolism, Amino sugar and nucleotide sugar metabolism, and Fructose and mannose metabolism. In contrast, the CSP group showed higher relative abundances of Citrate cycle (TCA cycle), Pyruvate metabolism, Propanoate metabolism, and Butanoate metabolism. Within Amino acid metabolism, the AHP group showed higher relative abundances of Arginine biosynthesis and Alanine, aspartate and glutamate metabolism, whereas the CSP group was enriched in Valine, leucine and isoleucine degradation, Tryptophan metabolism, Arginine and proline metabolism, Histidine metabolism, Lysine degradation, and Cysteine and methionine metabolism.

### 3.3 *In vitro* fermentation performance of reconstituted lyophilized rumen fluid

The *in vitro* fermentation performance of the reconstituted lyophilized rumen fluid is shown in Table 2. Compared with their respective substrate-free controls (AH-CON and CS-CON), the substrate-supplemented groups (AH and CS) exhibited significantly higher gas production (*p* < 0.0001), BCP (*p* = 0.0032), TVFA (*p* < 0.0001), acetate (*p* < 0.0001), propionate (*p* = 0.0122), and butyrate (*p* = 0.0002), along with significantly lower pH values (*p* < 0.0001). Between the two substrate-supplemented groups, the CS group showed significantly higher pH (*p* < 0.0001), acetate (*p* < 0.0001), isovalerate (*p* < 0.0001), TVFA (*p* < 0.0001), and A/P (*p* = 0.0015) than the AH group, whereas the AH group exhibited significantly higher gas production (*p* < 0.0001), butyrate (*p* = 0.0002), and valerate (*p* = 0.0026) than the CS group.

**Table 2:** *In vitro* fermentation performance of reconstituted lyophilized rumen fluid based on different roughage sources.

Items	AH	AH-CON	CS	CS-CON	*p*-value
Gas production (mL)	104.06 ± 1.29^a^	84.57 ± 3.57^c^	90.90 ± 4.14^b^	83.37 ± 4.09^c^	<0.0001
pH	4.28 ± 0.02^c^	4.53 ± 0.08^b^	4.48 ± 0.03^b^	4.58 ± 0.03^a^	<0.0001
NH_3_-N (mg/100 mL)	16.84 ± 0.76	16.12 ± 1.06	16.45 ± 1.05	16.27 ± 1.06	0.5056
BCP (mg/100 mL)	30.77 ± 2.15^a^	28.92 ± 1.53^b^	31.93 ± 1.89^a^	28.84 ± 1.52^b^	0.0032
TVFA (mmol/L)	42.75 ± 1.42^b^	38.72 ± 0.79^c^	46.25 ± 0.95^a^	42.48 ± 0.72^b^	<0.0001
Acetic (mmol/L)	31.95 ± 1.65^b^	28.71 ± 0.69^c^	35.90 ± 0.82^a^	32.87 ± 0.89^b^	<0.0001
Propionic (mmol/L)	6.24 ± 0.25^a^	5.72 ± 0.49^bc^	6.03 ± 0.12^ab^	5.58 ± 0.25^c^	0.0122
Butyric (mmol/L)	3.71 ± 0.11^a^	3.51 ± 0.11^b^	3.53 ± 0.12^b^	3.30 ± 0.11^c^	0.0002
Isobutyric (mmol/L)	0.17 ± 0.00	0.14 ± 0.01	0.15 ± 0.04	0.13 ± 0.01	0.1206
Valeric (mmol/L)	0.46 ± 0.01^a^	0.42 ± 0.01^ab^	0.40 ± 0.02^bc^	0.35 ± 0.08^c^	0.0026
Isovaleric (mmol/L)	0.23 ± 0.02^b^	0.22 ± 0.01^b^	0.26 ± 0.01^a^	0.25 ± 0.01^a^	<0.0001
A/P	5.13 ± 0.43^b^	5.06 ± 0.49^b^	5.96 ± 0.13^a^	5.91 ± 0.43^a^	0.0015

Values are presented as means ± SD (*n* = 9). VFA concentrations are expressed as mmol/L of fermentation fluid. AH, reconstituted pooled preparation of lyophilized rumen fluid based on an alfalfa hay diet with 1 g of substrate; AH-CON, the corresponding reconstituted preparation without substrate; CS, reconstituted pooled preparation of lyophilized rumen fluid based on a corn stover diet with 1 g of substrate; CS-CON, the corresponding reconstituted preparation without substrate. NH₃–N, ammonia nitrogen; BCP, bacterial crude protein; TVFA, total volatile fatty acids; A/P, acetate-to-propionate ratio. Values within the same row that do not share a common superscript letter differ significantly (*p* < 0.05).

## 4 Discussion

Lyophilization provides a critical strategy for stabilizing and standardizing inocula in RFT. In this study, the lyophilization yields of the AHP and CSP groups were 6.29 and 6.38%, respectively, with culturable carbohydrate-utilizing bacterial counts of 3.79 × 10^7^ and 3.93 × 10^7^ CFU/g. No significant intergroup differences were observed, suggesting that donor roughage source did not substantially affect lyophilization yield or culturable carbohydrate-utilizing bacterial counts in the resulting lyophilized rumen fluid. These similarities provided a broadly comparable basis for subsequent analyses. Yu et al. (2020) enriched ruminal microbial cells before lyophilization and obtained a yield of 2.50% and culturable carbohydrate-utilizing bacterial counts of approximately 6.8 × 10^8^ CFU/g. The absence of microbial enrichment in the present study may explain the higher yield and lower culturable counts, likely due to greater retention of soluble solids and nutrient substrates from the original rumen fluid. It should be noted that these selective culture-based counts represent only the fraction of bacteria capable of growth on the carbohydrate-containing medium under the specified conditions and therefore do not reflect total viable bacterial abundance or overall microbial survival after lyophilization. Overall, both lyophilized rumen fluid preparations contained substantial numbers of culturable carbohydrate-utilizing bacteria.

The VFA profiles were associated with the respective donor diets. In this study, under isoenergetic and isonitrogenous conditions, total VFA levels did not differ significantly between the AHP and CSP groups, but VFA composition differed markedly, indicating that roughage type primarily modulated the fermentation pattern rather than overall fermentation intensity. Previous studies have demonstrated that VFA production and composition are mainly governed by dietary chemical composition and nutrient supply characteristics (Guyader et al., 2017). The AHP group exhibited significantly elevated propionate and valerate concentrations. Compared with corn stover, alfalfa hay typically contains higher proportions of pectin and less cellulose. Pectin can be rapidly degraded by rumen microorganisms to promote the generation of fermentation intermediates (Bhandari et al., 2023). Zhang et al. (2014) reported that, compared with corn stover, alfalfa hay significantly increased the relative abundances of *Selenomonas* (a lactate-utilizing bacterium) and *Prevotella* (a pectin/hemicellulose-degrading bacterium) in the rumen, and that *Selenomonas* enrichment was thought to promote propionate accumulation through enhanced lactate utilization. *Prevotella* is one of the primary succinate-producing bacteria in the rumen, and succinate is an important precursor for propionate synthesis (Li Y. et al., 2023; El-Zaiat et al., 2026). These results suggest that alfalfa-based diets may promote propionate-related fermentation by altering rumen microbial community structure. Additionally, ruminal valerate can be derived from protein degradation or from carbohydrate fermentation intermediates such as lactate and succinate (Wang et al., 2024). In this study, the absence of significant changes in NH₃–N and BCP levels suggests that the elevated valerate in the AHP group was more likely linked to the fermentation of readily fermentable carbohydrates than to differences in protein degradation. By contrast, the higher acetate concentration and A/P in the CSP group may be attributable to the higher NDF level of the corn stover-based diet (45.13% vs. 35.61%). High-NDF diets fermented by fiber-degrading bacteria primarily yield acetate, with a concomitant rise in the A/P (Chen et al., 2008; Ma et al., 2022; Li et al., 2025). The CSP group also exhibited a higher isovalerate concentration. The primary source of ruminal isovalerate is dietary branched-chain amino acids, particularly leucine degradation (Zhan et al., 2023; Mota et al., 2025). Given the dietary composition in this study, protein in the corn stover-based donor diet was derived mainly from soybean and rapeseed meals, which are rich in leucine and thus supply ample substrates for isovalerate synthesis (Gołębiewska et al., 2022). Isovalerate has been reported as an important growth factor for cellulolytic bacteria such as *Ruminococcus* and *Fibrobacter* and is critical for maintaining ruminal fiber degradation function (Rezanejad et al., 2025). In summary, AHP was characterized by higher propionate and valerate levels, suggesting enhanced soluble fiber- and pectin-related fermentation, whereas CSP showed an acetate-dominant profile with higher isovalerate, consistent with structural carbohydrate fermentation.

The above fermentation phenotypes essentially represent the integrated outcomes of microbial community structure and metabolic interactions. In this study, bacterial, fungal, and methanogen community structures all differed significantly between the AHP and CSP groups, indicating that roughage type exerts a substantial influence on rumen microbial community structure. Additionally, the CSP group exhibited significantly higher Shannon diversity indices for both bacteria and fungi than the AHP group. This finding aligns with previous reports that high-NDF diets enhance rumen microbial diversity (Pitta et al., 2010; Zhang et al., 2014; Li et al., 2023a). Belanche et al. (2012) further demonstrated that fibrous diets significantly promote bacterial and fungal diversity through substrate heterogeneity and multi-stage degradation processes that foster synergistic interactions among distinct functional groups. In the present study, corn stover possesses higher NDF and ADF contents and a more complex fiber architecture than alfalfa hay, thereby providing more diverse ecological niches. Consequently, the elevated bacterial and fungal Shannon diversity observed in the CSP group likely reflects the influence of substrate heterogeneity on microbial richness and evenness.

At the bacterial level, the AHP group was significantly enriched in *Succinivibrio*, *Xylanibacter*, *Segatella*, and *Prevotellaceae_UCG-001*. Previous studies have demonstrated that alfalfa hay-based diets promote the adhesion, proliferation, and relative abundance of soluble carbohydrate- or plant polysaccharide-utilizing bacteria such as *Succinivibrio*, *Prevotella*, and *Prevotellaceae_UCG-001* in the rumen (Bian et al., 2024; Xie et al., 2025; Jackson et al., 2026). *Succinivibrio* is a typical succinate-producing bacterium that participates in soluble carbohydrate fermentation to generate succinate, acetate, and formate as products (Ren et al., 2019). *Xylanibacter* and *Segatella,* both formerly classified within *Prevotella*, together with *Prevotellaceae_UCG-001*, participate in hemicellulose, pectin, and other non-fibrous carbohydrate degradation through PULs and CAZyme systems, with acetate and succinate as their principal metabolites (Stewart et al., 2018; Liang et al., 2020). Therefore, the enrichment of these bacterial groups in the AHP group may enhance succinate-related metabolic potential. Given that succinate serves as an important precursor for propionate formation, this may partly explain the higher propionate concentration observed in the AHP group. Meanwhile, *Prevotellaceae_UCG-001* and *Segatella* were significantly positively correlated with propionate, and *Prevotellaceae_UCG-001*, *Succinivibrio*, *Segatella*, and *Xylanibacter* were all positively correlated with valerate, further suggesting that these AHP-enriched bacteria may participate in propionate- and valerate-related fermentation processes. By contrast, the CSP group was predominantly enriched in bacteria associated with structural carbohydrate degradation. *Rikenellaceae_RC9_gut_group* has been identified as an important hydrolytic and acidogenic genus during anaerobic fermentation of lignocellulosic biomass in the rumen (Zhang et al., 2025). *Ruminococcus* is a typical fiber-degrading bacterium capable of cellulose and hemicellulose degradation, with acetate as its principal fermentation end product (Liang et al., 2020). In this study, both *Rikenellaceae_RC9_gut_group* and *Ruminococcus* were positively correlated with pH and A/P, and negatively correlated with propionate and valerate; *Rikenellaceae_RC9_gut_group* was also positively correlated with acetate, suggesting that their enrichment may be associated with the structural carbohydrate degradation and higher A/P fermentation profile characteristic of the CSP group. The functions of *norank_f__F082* and *norank_f__[Eubacterium]_coprostanoligenes_group* remain incompletely characterized; however, the former exhibits variation patterns similar to those of fiber-degrading bacteria such as *Fibrobacter* and *Rikenellaceae_RC9_gut_group* (Zhou and Shen, 2025; Zhu et al., 2025), whereas the latter is enriched in environments with high dry matter digestibility (Liu et al., 2020). Combined with their positive correlations with A/P in this study, the enrichment of these genera may be associated with the structural carbohydrate-rich fermentation environment of the CSP group. Furthermore, the CSP group was enriched in genera associated with hemicellulose-derived carbon utilization. *norank_f__Bacteroidales_BS11_gut_group* can proliferate significantly under high-lignocellulose conditions and is considered capable of utilizing hemicellulose-derived monosaccharides to generate short-chain fatty acids (Solden et al., 2017). *Monoglobus* primarily utilizes pectin- and hemicellulose-derived uronic acids, pentoses (xylose and arabinose), and fructose, and maintains energy metabolism through acetate production (Kim et al., 2019). In this study, both *norank_f__Bacteroidales_BS11_gut_group* and *Monoglobus* were positively correlated with A/P and negatively correlated with propionate and valerate, with *Monoglobus* also significantly positively correlated with acetate, suggesting that these genera may be associated with hemicellulose-derived carbon utilization and the acetate-dominant fermentation phenotype of the CSP group. The enrichment of *norank_f__UCG-010* and *Candidatus Saccharimonas* likely reflects shifts in the CSP fermentation microenvironment. *norank_f__UCG-010* was significantly positively correlated with pH and A/P, and negatively correlated with propionate and valerate. Combined with reports that its abundance decreases in highly active fermentation environments (Zhou et al., 2023), this enrichment may be associated with microenvironments characterized by lower fermentation rates. Existing studies have shown that *Candidatus Saccharimonas* abundance varies with sodium acetate infusion, high-concentrate diets, low-energy high-fiber environments, and inflammation-associated microenvironments (Pang et al., 2022; Song et al., 2023; Zhen et al., 2023; Guo et al., 2024). In this study, this bacterium was significantly positively correlated with ruminal pH, acetate, A/P, and significantly negatively correlated with propionate and valerate. Therefore, this genus more likely serves as an indicator of fermentation microenvironmental shifts rather than performing specific substrate-degrading functions. Overall, the enrichment of bacteria utilizing soluble carbohydrates and plant polysaccharides in the AHP group aligns with its propionate- and valerate-associated fermentation characteristics, whereas the enrichment of fiber- and hemicellulose-utilizing bacteria in the CSP group corresponds to its acetate-dominant fermentation phenotype.

At the fungal level, the AHP group was significantly enriched in Neocallimastigomycota genera including *Neocallimastix*, *Piromyces*, and *Feramyces*. *Neocallimastigomycota* possess broad polysaccharide-degrading capabilities, secreting diverse CAZymes including cellulases, hemicellulases, pectinases, and various esterases and lyases, while their rhizoid systems mechanically penetrate plant cell walls to enhance the accessibility of internal polysaccharides (Solomon et al., 2016; Dhakal et al., 2025). Compared with corn stover, alfalfa hay exhibits lower lignification and higher contents of pectin and other plant polysaccharides, potentially providing a favorable substrate environment for fungal enrichment in the AHP group. *Piromyces* has been reported to be enriched under alfalfa hay-based diets (Wang R. et al., 2023). *Neocallimastix frontalis* and *Piromyces communis* can degrade xylan, cellulose, and pectic polysaccharides in alfalfa cell walls (Bootten et al., 2011). *Feramyces austinii F3a* can metabolize galactose, arabinose, alginate, and pectin (Hanafy et al., 2018). Additionally, the *Ascomycota* genus *Aspergillus* was significantly enriched in the AHP group. Certain *Aspergillus* species also possess pectinase and lignocellulase profiles and are associated with alfalfa hay intake (Tulsani et al., 2022; Li K. et al., 2024; Guo et al., 2025). These findings support the potential of AHP-enriched fungi to utilize pectin and other plant polysaccharides present in alfalfa hay. Correlation analysis further revealed that *Piromyces* and *Feramyces* were significantly positively correlated with propionate and valerate, while *Neocallimastix* and *Aspergillus* were significantly positively correlated with valerate; all these genera were negatively correlated with A/P. This correlation pattern aligns with the fermentation phenotype of the AHP group, suggesting that these fungi may degrade plant polysaccharides to provide accessible substrates for bacterial fermentation. By contrast, the CSP group was significantly enriched in multiple *Neocallimastigomycota* genera, including *Orpinomyces*, *unclassified_f__Neocallimastigaceae*, *Cyllamyces*, *Caecomyces*, *Pecoramyces*, *Anaeromyces*, and *Joblinomyces*. The enrichment of multiple anaerobic fungi in this group may be attributable to the higher cellulose, hemicellulose, and lignin contents of corn stover, reflecting the adaptive response of anaerobic fungi to complex stover-type fibrous substrates. Previous studies have identified *Orpinomyces*, *Neocallimastix*, *Caecomyces*, *Piromyces*, *Anaeromyces*, and *Cyllamyces* as important lignocellulose-degrading genera (Han et al., 2023). Notably, *Orpinomyces* sp. C1A exhibits strong cellulase and specific xylanase transcriptional responses on corn stover (Couger et al., 2015). *Caecomyces churrovis* efficiently utilizes corn stover as a substrate (Henske et al., 2017). Correlation analysis revealed that *Orpinomyces*, *Cyllamyces*, *Caecomyces*, *Anaeromyces*, *Joblinomyces*, *Pecoramyces*, and *unclassified_f__Neocallimastigaceae* were positively correlated with A/P; among these, *Cyllamyces*, *Caecomyces*, *Anaeromyces*, and *Joblinomyces* were also positively correlated with acetate. These results suggest that CSP-enriched anaerobic fungi may participate in the initial degradation of complex fibrous substrates, providing a substrate basis for acetate-dominant bacterial fermentation. Additionally, the *Ascomycota* genus *Alternaria* was significantly enriched in the CSP group; however, its functional role in ruminal fiber fermentation remains poorly defined, and its enrichment patterns are inconsistent across diverse hosts and dietary conditions (Ishaq et al., 2017; Wei et al., 2024). Therefore, it should not be directly interpreted as a genus functionally driving the CSP fermentation phenotype. Collectively, the fungal enrichment in the AHP group was oriented toward the utilization of pectin and plant polysaccharides in alfalfa hay, whereas the CSP group was enriched in multiple anaerobic fungal genera associated with complex fibrous substrate degradation. The divergence in fungal community composition between the two groups and the corresponding correlation patterns with fermentation parameters further support the conclusion that the roughage source in the donor diets influenced the fungal community structure and fermentation phenotype of the lyophilized rumen fluid.

At the methanogen level, the AHP group was significantly enriched in *Methanoculleus* and *Methanosphaera*. *Methanoculleus* is a hydrogenotrophic methanogen capable of producing methane from H₂, CO₂, and formate (Battumur et al., 2019). In this study, this genus was significantly positively correlated with propionate and valerate, and significantly negatively correlated with A/P, with correlation patterns consistent with the fermentation characteristics of the AHP group. Notably, *Succinivibrio,* enriched in the AHP group, can produce formate, which is also a methanogenic substrate utilizable by *Methanoculleus*. The significant positive correlation between these two genera in this study provides correlative support for a potential formate-mediated substrate linkage. *Methanosphaera* is a methylotrophic methanogen that primarily relies on H₂ to reduce methanol for methane production; ruminal methanol is mainly derived from pectin demethylation (Khairunisa et al., 2023). Peng et al. (2021), comparing multiple substrates, found that alfalfa selectively enriched *Methanosphaera stadtmanae* due to its higher pectin content. Li K. et al. (2024) also reported that alfalfa supplementation increased the abundance of this genus in the rumen. These studies indicate that the higher pectin content of alfalfa may promote methanol release, thereby providing a potential substrate source for *Methanosphaera* enrichment. In this study, *Methanosphaera* was significantly positively correlated with valerate, and significantly negatively correlated with acetate and A/P, suggesting that its enrichment aligns with the lower A/P fermentation environment characteristic of the AHP group. By contrast, the CSP group was enriched in *Methanobrevibacter* and *Methanolacinia*. *Methanobrevibacter* is a typical hydrogenotrophic methanogen that primarily produces methane via H₂-dependent CO₂ reduction (Khairunisa et al., 2023; Kang et al., 2025). Under high-fiber dietary conditions such as those with crop stover, *Methanobrevibacter* frequently emerges as the dominant methanogen (Xing et al., 2020; Xie et al., 2025). Wei et al. (2022) reported that methanogens naturally symbiotic with anaerobic fungi in the rumen of Qinghai yaks were exclusively *Methanobrevibacter*, and that this genus could enhance fiber degradation, acetate production, and methane generation in co-culture systems. In this study, *Methanobrevibacter* was also significantly positively correlated with A/P, cellulase activity, and CSP-enriched anaerobic fungi, suggesting that its enrichment may be associated with H₂ utilization, fiber degradation, and the acetate-dominant fermentation profile during high-fiber substrate fermentation in the CSP group. *Methanolacinia* was significantly positively correlated with pH, acetate, isovalerate, and A/P, and significantly negatively correlated with valerate, with its correlation pattern consistent with the higher A/P fermentation phenotype of the CSP group. However, functional reports on this genus in the rumen remain limited, and its ecological role requires further validation. Collectively, the enrichment of *Methanoculleus* and *Methanosphaera* in the AHP group likely reflects the substrate environment rich in pectin and soluble carbohydrates characteristic of alfalfa hay, whereas the enrichment of *Methanobrevibacter* in the CSP group aligns with high-fiber substrate fermentation and the elevated A/P fermentation phenotype.

Correlation analysis of intra- and inter-community relationships among bacteria, fungi, and methanogens in the lyophilized rumen fluid revealed that genera significantly enriched within each of the AHP and CSP groups were predominantly positively correlated with one another, suggesting that the roughage source in the donor diets was associated with distinct cross-domain microbial association patterns. Rumen fermentation typically relies on synergistic interactions among bacteria, fungi, and methanogens, including plant cell wall degradation, fermentation product transformation, and the transfer of reducing equivalents such as H₂ and formate (Cheng et al., 2009; Belanche et al., 2012; Han et al., 2023). Therefore, intra-group positive correlations may reflect synergistic adaptive relationships among different functional groups under the same diet environment. By contrast, enriched genera in the AHP and CSP groups were predominantly negatively correlated between groups. This pattern should not be interpreted as direct antagonism between the two microbial communities; rather, it more likely reflects community differentiation and alternative association patterns driven by differences in donor roughage compositions.

PICRUSt2 functional prediction further suggested potential metabolic differences between the AHP and CSP bacterial communities. The AHP group exhibited elevated relative abundances of Starch and sucrose metabolism, Fructose and mannose metabolism, Galactose metabolism, Amino sugar and nucleotide sugar metabolism, and Pentose and glucuronate interconversions, suggesting that its bacterial community possesses stronger potential for utilizing soluble sugars, pectin-related saccharides, and plant polysaccharide degradation products. This result aligns with the higher soluble carbohydrate and pectin contents of alfalfa hay, and corresponds to the enrichment of related bacterial groups such as *Prevotellaceae_UCG-001* and *Succinivibrio* in the AHP group, as well as its higher propionate and valerate concentrations. Concurrently, the AHP group showed elevated relative abundances of Arginine biosynthesis and Alanine, aspartate and glutamate metabolism. Arginine biosynthesis has been implicated in enhanced rumen nitrogen recycling (Fung et al., 2025). Alanine, aspartate and glutamate metabolism is closely linked to aminoacyl-tRNA synthesis, which provides substrates for BCP synthesis (Li Z. et al., 2024; Zhang et al., 2024; Sun et al., 2025). Therefore, the enrichment of these pathways in the AHP group suggests that this bacterial community may possess higher genomic metabolic potential for nitrogen assimilation and amino acid transformation. By contrast, the CSP group exhibited significantly higher relative abundances of Pyruvate metabolism, Citrate cycle (TCA cycle), Butanoate metabolism, and Propanoate metabolism. Pyruvate metabolism and the Citrate cycle (TCA cycle) serve as important hubs of central carbon metabolism, providing energy and biosynthetic precursors for cells (Prochownik and Wang, 2021). Their enrichment in the CSP group suggests that this bacterial community may possess greater potential for central carbon metabolism and energy conversion. Although the relative abundances of Butanoate metabolism and Propanoate metabolism were elevated in the CSP group, propionate and butyrate concentrations did not significantly increase, suggesting that these predicted pathways may more likely reflect the metabolic turnover of fermentation intermediates rather than directly corresponding to the accumulation of respective end products. This may be related to the further utilization or transformation of fermentation intermediates by specific microbial genera through cross-feeding in the gastrointestinal tract (Sheridan et al., 2022). Furthermore, the CSP group showed significantly elevated relative abundances of Valine, leucine and isoleucine degradation, Lysine degradation, and multiple amino acid metabolism-related pathways. Amino acid degradation can provide gastrointestinal microorganisms with additional energy, nitrogen sources, and carbon skeletons entering central metabolism (Van den Abbeele et al., 2022). Notably, enrichment of the Valine, leucine and isoleucine degradation pathway suggests higher BCAA degradation potential in the CSP bacterial community, which together with the aforementioned BCAA substrate supply may provide a metabolic basis for isovalerate production, consistent with the elevated isovalerate levels in this group. Therefore, the CSP bacterial community may exhibit greater potential for amino acid degradation and protein-derived substrate utilization. This likely reflects an enhanced genomic potential for utilizing carbon skeletons and nitrogen sources from amino acids under conditions where structural carbohydrates constitute a higher proportion and readily available carbon sources are relatively limited. Collectively, PICRUSt2 functional predictions indicate that the AHP bacterial community is oriented toward the utilization of soluble sugars, pectin-related saccharides, and plant polysaccharide degradation products, and may possess higher nitrogen assimilation and amino acid transformation potential. The CSP group, conversely, may have greater potential for central carbon metabolism, amino acid degradation, and high-fiber substrate fermentation-related metabolism. It should be emphasized that PICRUSt2 functional predictions are based on 16S rRNA gene data and reference genomes and therefore reflect only genomic metabolic potential rather than actual gene expression, enzyme activity, or metabolic flux.

By reconstituting lyophilized rumen fluid from the two donor-diet groups, this study further evaluated the substrate fermentation capacity using *in vitro* fermentation assays. Compared with their corresponding substrate-free controls, the AH and CS groups with added fermentation substrate exhibited significantly higher gas production, BCP, TVFA, acetate, propionate, and butyrate concentrations, along with significantly lower pH. These results indicate that the lyophilized rumen fluid exhibited basal fermentative activity upon reconstitution, including acid production and BCP synthesis, and effectively utilized exogenous substrates. The substrate-free control groups (AH-CON and CS-CON) still produced measurable gas, likely attributable to residual fermentable components and endogenous microbial substrates in the lyophilized rumen fluid. This finding indicates that the reconstituted preparations exhibited basal metabolic activity, while exogenous substrate addition further enhanced fermentation activity. Chaudhry and Mohamed (2012) froze total rumen contents at −20 °C for 4 weeks and then used them for *in vitro* substrate fermentation, finding that frozen rumen contents retained substrate fermentation capacity, with feed degradation effects highly correlated between frozen and fresh rumen contents (*R^2^* ≥ 0.95). These results indicate that although low-temperature storage may affect the activity of certain microorganisms, the fermentative functions of rumen microbiota can be maintained to a certain extent. Following reconstitution, distinct differences in *in vitro* substrate fermentation characteristics were observed between the AH and CS treatments. The CS group exhibited significantly higher pH, acetate, isovalerate, TVFA, and A/P than the AH group. Conversely, the AH group showed significantly higher gas production, butyrate, and valerate than the CS group, although propionate concentrations were numerically higher without reaching statistical significance. The differences in fermentation profiles between the AH and CS groups generally paralleled those observed between the corresponding AHP and CSP groups, suggesting that rumen microbial fermentation characteristics associated with the different roughage sources in the donor diets remained evident, at least in part, after lyophilization and reconstitution. Mota et al. (2024) conducted *in vitro* fermentation using fresh rumen fluid collected from animals fed different diets and found that rumen fluid from high-fiber diets reduced gas production while increasing fermentation pH and the acetate proportion, a trend consistent with the present results. Zhang et al. (2022) reported that substrate fermentability is typically associated with increased gas production and TVFA generation. In the present study, although the AH group exhibited lower TVFA than the CS group, its gas production was significantly higher, indicating that gas production and VFA accumulation are not entirely concordant. This discrepancy may be related to differences in substrate fermentation pathways, gas generation ratios, and the dynamics of VFA production and utilization. This study also found that the AH group exhibited significantly higher butyrate concentrations during *in vitro* fermentation than the CS group, a difference that was not observed between the corresponding AHP and CSP groups. Previous studies have confirmed that ruminal butyrate production is positively correlated with soluble fiber content (Hoyles et al., 2018; Du et al., 2025). In summary, lyophilized rumen fluid from both donor-diet groups exhibited microbial fermentative activity upon reconstitution and effectively utilized exogenous substrate during *in vitro* fermentation. The AH group primarily exhibited characteristics associated with soluble carbohydrate fermentation, whereas the CS group exhibited an acetate-dominant fermentation phenotype consistent with a high-fiber donor-diet background.

Several limitations should be considered. First, because fresh rumen fluid controls were not included, the extent to which fermentative function and donor-diet-related characteristics were retained after lyophilization and reconstitution could not be determined. The *in vitro* assay showed that the reconstituted preparations produced gas and fermentation products and that the AH and CS groups exhibited differences broadly consistent with their respective donor diets; however, these results do not quantify the degree of preservation. Second, although gas production was monitored throughout the 24-h incubation, pH, fermentation metabolites, and BCP were measured only at the end of incubation; therefore, their temporal changes were not evaluated. Third, the present findings characterize the microbial composition of the lyophilized rumen fluid and the *in vitro* fermentation performance of the reconstituted preparations but do not provide direct evidence of their efficacy following transplantation into animals. Finally, although the donor goats originated from a farm with an established animal-health and biosecurity program and were confirmed seronegative for brucellosis before rumen fluid collection, the final lyophilized preparations were not directly screened for specific pathogens. Therefore, the pathogen-free status of individual batches could not be established. Before use in animal transplantation studies or practical livestock production, each batch should undergo validated screening for *Brucella* spp., foot-and-mouth disease virus, and other pathogens relevant to local epidemiological risks.

## 5 Conclusion

This study demonstrated that the roughage source in the donor diets did not significantly affect the lyophilization yield or culturable carbohydrate-utilizing bacterial counts, but significantly influenced the bacterial, fungal, and methanogen community structures of the lyophilized rumen fluid. Moreover, the different roughage sources in the donor diets were associated with distinct cross-domain microbial association patterns that were consistent with the observed differences in fermentation phenotypes. Lyophilized rumen fluid based on the alfalfa hay diet exhibited higher propionate and valerate concentrations, and its microbial community was characterized by the enrichment of genera associated with the utilization of soluble carbohydrates, pectin, and plant polysaccharide degradation products. In contrast, lyophilized rumen fluid based on the corn stover diet exhibited higher acetate and isovalerate concentrations, a higher A/P, and higher bacterial and fungal Shannon indices, and its microbial community was characterized by the enrichment of taxa associated with structural carbohydrate degradation. After reconstitution, both preparations effectively utilized exogenous substrates during *in vitro* fermentation and showed fermentation patterns generally consistent with those associated with their respective donor diets. Overall, lyophilized rumen fluid may serve as a readily storable inoculum with potential for standardization in RFT. These findings provide a basis for further evaluation and optimization of RFT strategies for modulating rumen microbial ecology in ruminants.

## Data Availability

The raw sequencing data presented in this study have been deposited in the NCBI Sequence Read Archive (SRA) under BioProject accession number PRJNA1469355. Other data are available from the corresponding author upon reasonable request.

## Footnotes

1
https://v.majorbio.com
