# Supplementary material for: Lyophilized rumen fluid from goats fed different roughage-based diets: fermentation characteristics, microbial communities, and post-reconstitution in vitro fermentation performance
Source: Front Microbiol. 2026 Sep 15;17:1905439. doi: 10.3389/fmicb.2026.1905439 (PMC13619837; doi:10.3389/fmicb.2026.1905439)
Supplement: Supplementary file 1 [file Supplementary_file_1.docx]

**Supplementary Material**

**Methods S1. Detailed procedures for primer sequences, PCR amplification, and library preparation**

**Primer sequences.** The V3–V4 region of the bacterial 16S rRNA gene was amplified using primers 338F (5′-ACTCCTACGGGAGGCAGCAG-3′) and 806R (5′-GGACTACHVGGGTWTCTAAT-3′). The fungal ITS1 region was amplified using primers ITS1F (5′-CTTGGTCATTTAGAGGAAGTAA-3′) and ITS2R (5′-GCTGCGTTCTTCATCGATGC-3′). The methanogen mcrA gene was amplified using primers MLf (5′-GGTGGTGTMGGATTCACACARTAYGCWACAGC-3′) and MLr (5′-TTCATTGCRTAGTTWGGRTAGTT-3′).

**PCR amplification.** Each PCR reaction was performed in a total volume of 20 μL containing 4 μL of 5× TransStart FastPfu buffer, 2 μL of 2.5 mM dNTPs, 0.8 μL each of forward and reverse primers (5 μM), 0.4 μL of TransStart FastPfu DNA polymerase, and 10 ng of template DNA, with the final volume adjusted to 20 μL using ddH₂O. PCR amplification was performed using an ABI GeneAmp® 9700 thermal cycler (Applied Biosystems, Foster City, CA, USA). The thermal cycling program consisted of initial denaturation at 95 °C for 3 min; 27 cycles of denaturation at 95 °C for 30 s, annealing at 55 °C for 30 s, and extension at 72 °C for 30 s; followed by final extension at 72 °C for 10 min.

**Library preparation.** Amplified products were examined by 2% agarose gel electrophoresis, purified using a PCR Clean-Up Kit (Yuhua, China), and quantified using a Qubit 4.0 fluorometer (Thermo Fisher Scientific, Waltham, MA, USA). Sequencing libraries were constructed using the NEXTFLEX Rapid DNA-Seq Kit and sequenced on an Illumina NextSeq 2000 platform.

**Additional bioinformatic tools.** Community structures were visualized using bar plots and heatmaps.

**Table S1.** Composition and nutrient levels of the experimental diets (DM basis, %)

| Ingredients | Alfalfa hay group | Corn stover group | Nutrient Levels | Alfalfa hay group | Corn stover group |
| --- | --- | --- | --- | --- | --- |
| Corn stalk | 0.00 | 50.00 | CP | 13.23 | 13.13 |
| Alfalfa hay | 50.00 | 0.00 | NDF | 35.61 | 45.13 |
| Corn | 28.20 | 26.50 | ADF | 20.79 | 27.82 |
| Rapeseed meal | 0.00 | 5.00 | EE | 3.68 | 3.67 |
| Soybean meal | 0.00 | 15.10 | Starch | 20.35 | 20.47 |
| Corn husk | 12.50 | 0.00 | ME² (MJ/kg) | 12.03 | 12.05 |
| Wheat bran | 6.00 | 0.00 |  |  |  |
| Soybean oil | 0.30 | 0.40 |  |  |  |
| Salt | 0.50 | 0.50 |  |  |  |
| Premix^1^ | 2.50 | 2.50 |  |  |  |
| Total | 100.00 | 100.00 |  |  |  |

CP, crude protein; NDF, neutral detergent fiber; ADF, acid detergent fiber; EE, ether extract; ME, metabolizable energy.

^1^ The premix contained/kg diet: vitamin A 6 000 IU, vitamin D3 2000 IU, vitamin E 15 IU, vitamin K3 1.8 mg, vitamin B1 0.35 mg, vitamin B2 8.5 mg, vitamin B6 0.9 mg, vitamin B12 0.03 mg, D-pantothenic acid 16 mg, nicotinic acid 22 mg, folic acid 1.5 mg, biotin 0.15 mg, Cu 8 mg, Fe 40 mg, Mn 20 mg, Zn 40 mg, I 0.8 mg, Se 0.3 mg, Co 0.3 mg. ^2^ ME was calculated value, while others were all measured values.

**Table S2.** Composition and nutrient levels of the in vitro fermentation substrate (DM basis, %)

| Ingredient | Content | Nutrient composition | Content |
| --- | --- | --- | --- |
| Alfalfa hay | 25.00 | CP | 13.31 |
| Corn stover | 25.00 | NDF | 40.30 |
| Corn | 27.50 | ADF | 24.38 |
| Rapeseed meal | 2.50 | EE | 3.76 |
| Soybean meal | 7.85 | Starch | 20.42 |
| Corn husk | 6.25 | ME² (MJ/kg) | 12.17 |
| Wheat bran | 3.00 |  |  |
| Soybean oil | 0.40 |  |  |
| Salt | 0.50 |  |  |
| Premix^1^ | 2.00 |  |  |
| Total | 100.00 |  |  |

CP, crude protein; NDF, neutral detergent fiber; ADF, acid detergent fiber; EE, ether extract; ME, metabolizable energy.

^1^ The premix contained/kg diet: vitamin A 6 000 IU, vitamin D3 2000 IU, vitamin E 15 IU, vitamin K3 1.8 mg, vitamin B1 0.35 mg, vitamin B2 8.5 mg, vitamin B6 0.9 mg, vitamin B12 0.03 mg, D-pantothenic acid 16 mg, nicotinic acid 22 mg, folic acid 1.5 mg, biotin 0.15 mg, Cu 8 mg, Fe 40 mg, Mn 20 mg, Zn 40 mg, I 0.8 mg, Se 0.3 mg, Co 0.3 mg. ^2^ ME was calculated value, while the others were measured values.
